# Exploration of macromolecular phenotype of human skeletal muscle in diabetes using infrared spectroscopy

**DOI:** 10.3389/fendo.2023.1308373

**Published:** 2023-12-21

**Authors:** Barbara Zupančič, Chiedozie Kenneth Ugwoke, Mohamed Elwy Abdelhamed Abdelmonaem, Armin Alibegović, Erika Cvetko, Jože Grdadolnik, Anja Šerbec, Nejc Umek

**Affiliations:** ^1^ Laboratory for Molecular Structural Dynamics, Theory Department, National Institute of Chemistry, Ljubljana, Slovenia; ^2^ Institute of Anatomy, Faculty of Medicine, University of Ljubljana, Ljubljana, Slovenia; ^3^ Biotechnical Faculty, University of Ljubljana, Ljubljana, Slovenia; ^4^ Department of Forensic Medicine and Deontology, Faculty of Medicine, University of Ljubljana, Ljubljana, Slovenia

**Keywords:** diabetes mellitus, skeletal muscle, metabolism, macromolecular composition, infrared spectroscopy, multivariate analysis, histochemical assays

## Abstract

**Introduction:**

The global burden of diabetes mellitus is escalating, and more efficient investigative strategies are needed for a deeper understanding of underlying pathophysiological mechanisms. The crucial role of skeletal muscle in carbohydrate and lipid metabolism makes it one of the most susceptible tissues to diabetes-related metabolic disorders. In tissue studies, conventional histochemical methods have several technical limitations and have been shown to inadequately characterise the biomolecular phenotype of skeletal muscle to provide a holistic view of the pathologically altered proportions of macromolecular constituents.

**Materials and methods:**

In this pilot study, we examined the composition of five different human skeletal muscles from male donors diagnosed with type 2 diabetes and non-diabetic controls. We analysed the lipid, glycogen, and collagen content in the muscles in a traditional manner with histochemical assays using different staining techniques. This served as a reference for comparison with the unconventional analysis of tissue composition using Fourier-transform infrared spectroscopy as an alternative methodological approach.

**Results:**

A thorough chemometric post-processing of the infrared spectra using a multi-stage spectral decomposition allowed the simultaneous identification of various compositional details from a vibrational spectrum measured in a single experiment. We obtained multifaceted information about the proportions of the different macromolecular constituents of skeletal muscle, which even allowed us to distinguish protein constituents with different structural properties. The most important methodological steps for a comprehensive insight into muscle composition have thus been set and parameters identified that can be used for the comparison between healthy and diabetic muscles.

**Conclusion:**

We have established a methodological framework based on vibrational spectroscopy for the detailed macromolecular analysis of human skeletal muscle that can effectively complement or may even serve as an alternative to histochemical assays. As this is a pilot study with relatively small sample sets, we remain cautious at this stage in drawing definitive conclusions about diabetes-related changes in skeletal muscle composition. However, the main focus and contribution of our work has been to provide an alternative, simple and efficient approach for this purpose. We are confident that we have achieved this goal and have brought our methodology to a level from which it can be successfully transferred to a large-scale study that allows the effects of diabetes on skeletal muscle composition and the interrelationships between the macromolecular tissue alterations due to diabetes to be investigated.

## 1 Introduction

Diabetes mellitus (DM) has become a global epidemic, representing a significant public health challenge in the 21^st^ century with a staggering burden on both healthcare systems and individuals (1, 2). According to the International Diabetes Federation (IDF), 537 million adults (20-79 years) had DM in 2021, and this number is expected to increase to 643 million by 2030. In 2021, DM caused 6.7 million deaths (3). These worrisome realities have prompted a global consensus to halt the rise in diabetes and obesity by 2025 (1), highlighting the urgent need for more effective preventive and therapeutic strategies based on a better understanding of the pathophysiological mechanisms underlying the disease. However, the multifactorial nature of DM, including its complex aetiology and diverse clinical manifestations, presents considerable challenges for diagnosis and treatment.

Among the various organs affected, skeletal muscles play a critical role in the pathophysiology and metabolic complications of DM (4–10). Comprising approximately 40% of the total body mass in humans, skeletal muscles are major contributors to whole-body glucose homeostasis and energy expenditure. As the largest endocrine tissue involved in glucose metabolism, it mediates about 80% of insulin-stimulated glucose uptake (11). Decreased sensitivity for this uptake in skeletal muscle contributes to whole-body metabolic dysregulation and cardiovascular risk and is a core pathophysiological factor in several metabolic phenotypes (12, 13). Skeletal muscles are structurally composed of multiple fascicles or bundles of physiochemically and metabolically distinct fibre types, which are classified based on the expression of different isoforms of the myosin heavy chain (14, 15). Depending on their oxidative and glycolytic capacity, healthy skeletal muscles can rapidly switch between carbohydrate and lipid fuels according to bioenergetic demand. Loss of this flexibility is one of the hallmarks of metabolic diseases (16).

The pathobiochemical changes in diabetic muscle remain a focal point of several studies aiming to clarify the molecular and cellular mechanisms of insulin resistance in diabetes and associated clinical complications. For instance, proteomic profiling of skeletal muscle from diabetic animal models and diabetic human skeletal muscle have been considered in an attempt to identify protein factors to monitor diabetic progression (17). The study by Öhman et al. (18) on the skeletal muscle proteome in biopsies of vastus lateralis muscle showed altered phosphorylation in several signalling pathways in impaired fasting glucose, impaired glucose tolerance and type 2 DM. Gilbert reviewed the role of skeletal muscle lipids in the pathogenesis of insulin resistance in obesity and type 2 DM, but left unanswered the question of whether fatty acids are causative molecular players or markers of reduced insulin sensitivity (19). It has been shown that changes in glucose transport activity appear to stem from disruptions in intramyocellular fatty acid metabolism, where fatty acids induce insulin resistance through a serine kinase cascade activation, resulting in reduced tyrosine phosphorylation of IRS-1 and diminished IRS-1-associated phosphatidylinositol 3-kinase activity, which is crucial for insulin-mediated glucose transport in muscle (20). Most studies agree that it is not intramuscular lipids per se that cause insulin resistance, but rather lipid intermediates such as diacylglycerols, fatty acyl-CoAs and ceramides, and that it is the localisation, composition and turnover of these intermediates that play an important role in the development of insulin resistance and type 2 DM (21–23). Nevertheless, lipid accumulation in skeletal muscle cells is both muscle and fibre-type specific. As shown by Umek et al. (15), intramyocellular lipid accumulation was most pronounced in type 2a and 2x/d fibres of fast-twitch gastrocnemius and intermediate plantaris muscles in obese insulin-resistant mice compared to lean mice, whereas no significant lipid accumulation was observed in the slow-twitch soleus muscle in the obese animals. He et al. (24) reported higher lipid content within each fibre type of muscles from obese and type 2 diabetic subjects. The review by Chang et al. (25) provides insight into the experimental and clinical studies implicating the role of phospholipids in a diverse range of physiological processes, including their role as critical mediators of insulin action on skeletal muscle.

In type 2 DM, a reduction of muscle glycogen was observed, where the deficit was marked in type IIa (fast oxidative) fibres, which make up almost 50% of muscle fibres in vastus lateralis, and was minor in types I and IIb (slow oxidative and fast glycolytic, respectively) fibres (26). A key novel finding by Frankenberg et al. was that the majority of glycogen in human skeletal muscle is loosely bound or cytosolic. The proportion of this diffusible glycogen pool was significantly lower in the type I fibres in type 2 diabetic muscle compared to the control group, whereby the hyperinsulinemic clamp in people with type 2 diabetes had no effect on the proportion of diffusible glycogen (27). In addition, increased collagen content has been identified as a hallmark of insulin-resistant skeletal muscle in overweight and type 2 diabetic individuals. Immunofluorescence staining of muscle biopsies showed increased abundance of type I and III collagen (28–30).

Molecular, histochemical, and immunohistochemical assays are the most commonly employed methods to investigate skeletal muscle changes in DM and other metabolic disorders. However, these techniques have several drawbacks, including the inability to provide a comprehensive molecular profile of the alterations occurring in the tissue and reliance on methods that may introduce subjective interpretation biases (31–33). To overcome these challenges, there is a need for advanced analytical techniques with improved quantitative capabilities, efficiency and objectivity.

Vibrational spectroscopy techniques, such as Fourier-transform infrared (FTIR) spectroscopy, have emerged as powerful tools that can provide a reliable and promising alternative for the analysis of biological tissues (34, 35). The spectra obtained provide highly informative data on tissue composition via characteristic absorption bands that can be used to identify and quantify different macromolecules within a sample. FTIR has the advantage of rapidness and high molecular specificity with minimal sample preparation compared to histochemical analyses (33, 36). This allows sensitive detection of biomolecular changes in many functional groups simultaneously (37). The technique has been successfully applied in diverse biological tissue analysis. For example, for the diagnosis of cutaneous neoplasia and the detection of carcinogenesis-associated bimolecular changes (38–43), for the classification of different subtypes of cancer (44) and for prediction of metastatic behaviour (45, 46). Other examples of the successful application of infrared (IR) spectroscopy include the detection of DM-induced lipid peroxidation in rat liver microsomal membranes (47), noninvasive estimation of blood haemoglobin A1c (HbA1c) levels (48), investigation of biochemical and structural changes in neurodegenerative diseases (49, 50), and other tissue dysfunctions (51–54). Regarding diabetes, the review by Ralbovsky and Lednev summarises the recent applications of vibrational spectroscopy in DM diagnostic research (55). In particular, the potential of FTIR spectroscopy for investigating of various tissues for novel biomarkers in diabetes has been reported, e.g. considering tissues such as blood (56), saliva (57–59), urine (60), bone (61, 62), pulmonary oedema fluid (63). On the other hand, there are studies presenting the use of FTIR to analyse molecular profiles of muscle tissue in animal models (64–66) and human muscle (67, 68). Nevertheless, there are only a few studies that have used FTIR spectroscopy to investigate modifications in muscle tissue associated with DM, and even these have been performed in animal models (69–71).

Our recently published study also focused on the detection of changes in skeletal muscle composition due to obesity with insulin resistance and STZ-induced diabetes in a mouse model using FTIR (72). Building on the lessons and outcomes of this study, in the present work, we aim to transition from our research on metabolic disorders affecting skeletal muscle tissue in animal models and investigate the macromolecular composition of different human skeletal muscles in type 2 DM. In this context, we present an improved methodology that combines FTIR experiments and powerful chemometric tools to study wet samples from five functionally and histologically diverse human skeletal muscles obtained from diabetic and non-diabetic male donors. The improved approach to analysing the FTIR spectra involves a multi-stage decomposition of the IR spectra into several spectral components related to the vibrational properties of the particular biomolecular constituents of skeletal muscle, allowing a detailed characterisation of their composition. We would like to emphasise that this is a pilot study whose main purpose is primarily to establish a solid methodological background for an in-depth analysis of the composition of healthy and diabetic human muscles based on vibrational spectroscopy. With this, we aim to have available a versatile and efficient experimental-chemometric tool for the large-scale study of diabetes-related alterations in skeletal muscle composition in the subsequent of our research.

## 2 Materials and methods

The study was conducted in accordance with the Declaration of Helsinki. The protocols for the use of human skeletal muscle tissue were reviewed and approved by the Republic of Slovenia National Medical Ethic Committee (Permit No.: 0120-536/2019/4).

### 2.1 Donors and skeletal muscle samples

**Figure 1:**
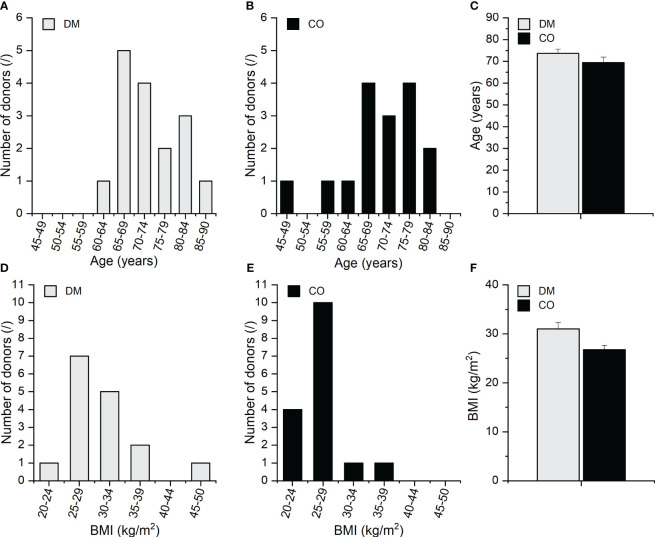
Age distribution of the **(A)** DM and **(B)** CO donors; **(C)** average age of the DM and CO donors, body mass index (BMI) distribution of the **(D)** DM and **(E)** CO donors; **(F)** average BMI of the DM and CO donors. Data in **(C, F)** are means with standard errors of the mean (SEM).

Skeletal muscle tissue samples were harvested from 32 deceased adult (≥ 18 years) male bodies, including 16 diabetic (further abbreviated as diabetic group DM) and 16 non-diabetic donors (further abbreviated as control group CO) within 24 hours postmortem during standard autopsy procedures at the Institute of Forensic Medicine, University of Ljubljana, Slovenia. The age structure and body mass index (BMI) of the 16 diabetic male donors and 16 non-diabetic male donors are presented in **Figure 1**; **Supplementary Figure 1** shows their scatterplots. The data are for information only, and their effects were not analysed in this pilot work but will be considered in the scale-up study with larger statistical samples in the next phase of our research. Type 2 DM status was established based on clinical and biochemical evidence from the patients’ medical records. All diabetic patients included had a history of treatment with both oral antidiabetic medications and insulin. Subjects with type 1 DM and other morbidities or recent history of therapy that is known to influence skeletal muscle fibre typology and biochemical composition (e.g. genetic myopathies, chronic heart failure, chronic obstructive pulmonary disease, glucocorticoid treatment, etc.) were excluded. The non-diabetic donors consist of male subjects with no history of diabetes or other conditions known to specifically impact skeletal muscle phenotype. For non-diabetic controls, DM or related glycaemic phenotypes were ruled out based on a thorough examination of the donors’ medical records and additionally confirmed with a normal postmortem result for the glycated haemoglobin (HbA1c), which shows the glycaemic status in the preceding two months. The donors’ basic clinical data, including age, body mass, and height, were recorded, and the obtained samples and data were pseudonymised.

Five different skeletal muscles, including axial, appendicular, and respiratory muscles, were harvested, i.e. levator scapulae muscle (LEV), splenius capitis muscle (SPL), vastus lateralis muscle (VL), diaphragm (DIA), and external intercostal muscles (EXT). Given the structural, metabolic, and functional diversity of human skeletal muscles, a differential effect of DM on the phenotype of skeletal muscles in different anatomical regions is expected (73). Accordingly, these five functionally distinct skeletal muscles were selected to better understand the heterogeneous effects of DM on various skeletal muscle types. The VL is a commonly studied limb muscle in humans due to its easy accessibility and is, therefore, a reference muscle for biomedical investigations. However, like most locomotor muscles, physical activity status may influence its metabolic phenotype and confound disease-attributable effects (74). The EXT and DIA are respiratory muscles metabolically adapted to sustain continuous activity during life (75). The axial skeletal muscles, such as the LEV and the SPL, are less commonly investigated due to the difficulty of clinical biopsies but may be less profoundly impacted by physical activity than limb muscles. The DIA muscle was harvested from the mid-portion of the muscle along the right midclavicular line; EXT from the sixth intercostal space along the right midclavicular line; LEV from the superior aspect of the left muscle, beneath the superior aspect of the sternocleidomastoid (around C3/4 vertebral levels); SPL from the mid-portion of the left muscle, at the C4 vertebral level, along the plane of the fibres running superolaterally from the proximal attachment at the nuchal ligament to the distal attachment at the mastoid process of the left temporal bone; and VL from the mid-portion of the left muscle, midway between the greater trochanter of the femur and the upper border of the patella. Samples were immediately processed, frozen in liquid nitrogen, and stored at -80°C until sectioning for histochemical assays and spectroscopic measurements.

### 2.2 FTIR spectroscopy experiments

FTIR spectroscopy measurements were performed in attenuated total reflection (ATR) mode on the Bruker Vertex 80 spectrometer. For ATR sampling, the Specac Golden Gate accessory with a single reflection diamond was used. The temperature of the ATR cell was kept constant at 24°C using the temperature controller. The spectrometer optics and the ATR cell were purged with technical dry nitrogen during the measurements. One ATR-FTIR spectrum was measured for each specimen by averaging 128 interferograms in the range 4000 cm^-1^–600 cm^-1^ with a spectral resolution of 2 cm^-1^ using the software OPUS, version 7.8 (Bruker, Billerica, MA, USA).

One hundred and sixty (160) wet skeletal muscle specimens (5 different muscles from 32 donors) for FTIR measurements were prepared in the form of several 20 μm cryosections with a mass of about 1 mg and stored in microcentrifuge tubes at -20°C before the experiments. The frozen tissue specimen was placed on the ATR cell, and the temperature was allowed to stabilize. In the meantime, it was thoroughly kneaded with a spatula to homogenize the tissue constituents. It was then pressed against the ATR crystal to ensure good contact between the crystal and the tissue. In addition to the spectroscopic measurements of the muscle tissue, the ATR-FTIR spectrum of water was also recorded every time to serve as a reference to delineate the vibrational properties of water and macromolecular components of the wet tissue when analysing spectral data. It should be noted that for certain samples, it was not possible to mechanically remove all the adipose tissue during sample preparation. Therefore, in these cases, the measured FTIR spectra reflected not only the vibrational properties of the skeletal muscle but also those of the adipose tissue. We took this into account and managed to separate the vibrational spectrum of the adipose tissue from the spectra of the skeletal muscle by analysing the spectra chemometrically using a multi-level spectral decomposition, as explained in the sequel.

### 2.3 FTIR spectral data analysis

The OPUS software version 7.8 (Bruker, Billerica, MA, USA) was used for compensation of atmospheric water and CO_2_ and for baseline correction of the measured ATR-FTIR spectra of tissue samples and water. No ATR correction was performed. The water spectra measured each time we performed experiments were averaged, and normalisation factors were calculated based on the intensity of the OH stretching peak relative to the intensity of the OH stretching peak of the average water spectrum. These normalisation factors were further used to normalise the ATR-FTIR spectra of skeletal muscles measured on the same occasion as the corresponding water spectrum. The normalised ATR-FTIR spectra (abbreviated as NS in the following) are presented in **Supplementary Figure 2** in the form of average spectrum and the dispersion of the spectra around the average.

We then subjected the normalised spectra NS to multivariate curve resolution decomposition with alternating least square optimization (MCR-ALS decomposition) using MATLAB software *MCR-ALS GUI v4c* (76, 77). The decomposition was performed in three steps, where in the first decomposition the spectral component AD was identified as the one reflecting the vibrational spectrum of the adipose tissue. This component was subtracted from the spectra to treat only the spectra representing the vibrational properties of skeletal muscle tissue in the subsequent steps of the decomposition. The second MCR decomposition step resulted in the spectral component MC3 and after its subtraction from the spectra, we obtained the spectral components MC1 , MC2 and  BW in the third MCR decomposition step. For each decomposition step, more than 99% of the total variance was explained. Thus, after the three-step decomposition, the normalised spectra NS from the experiments were split into several spectral components as follows.


$$\begin{array}{l} {\;}_{\text{muscle}}\text{N}{\text{S}}_{\text{group},\text{i}}{=}_{\text{muscle}}\text{c}1_{\text{group},\text{i}}\cdot \text{MC}1{+}_{\text{muscle}}\text{c}2_{\text{group},\text{i}}\cdot \text{MC}2\\ {+}_{\text{muscle}}\text{c}3_{\text{group},\text{i}}\cdot \text{MC}3+\text{a}{\;}_{\text{di}}\cdot \text{AD}+\text{b}{\text{w}}_{\text{i}}\cdot \text{BW}{+}_{\text{muscle}}{\text{r}}_{\text{group},\text{i}} \end{array}$$

for


(1) $$\begin{array}{c} \text{muscle}\in \left\{\text{DIA},\text{EXT},\text{LEV},\text{SPL},\text{VL}\right\}\\ \text{group}\in \left\{\text{DM},\text{CsO}\right\}\\ \text{i}\in \left\{1,2,\ldots 16\right\} \end{array}$$

The spectral component BW in Eq. (1) represents the body water content in skeletal muscle and was not analysed further. In addition, the component AD , representing adipose tissue, was considered a side result due to sample preparation limitations to completely remove adipose from skeletal muscle.

Eq. (2) represents the vibrational spectra  MmuscleCgroup,i as obtained after removing the spectral components of body water BW and adipose tissue AD from the normalised ATR-FTIR spectra,  NmuscleSgroup,i from Eq. (1), i.e.,


$$\begin{array}{c} {\;}_{\text{muscle}}\text{M}{\text{C}}_{\text{group},\text{i}}{=}_{\text{muscle}}\text{N}{\text{S}}_{\text{group},\text{i}}-\text{b}{\;}_{\text{wi}}\cdot \text{BW}-\text{a}{\;}_{\text{di}}\cdot \text{AD}\\ {=}_{\text{muscle}}\text{c}1_{\text{group},\text{i}}\cdot \text{MC}1{+}_{\text{muscle}}\text{c}2_{\text{group},\text{i}}\cdot \text{MC}2{+}_{\text{muscle}}\text{c}3_{\text{group},\text{i}}\cdot \text{MC}3{+}_{\text{muscle}}{\text{r}}_{\text{group},\text{i}} \end{array}$$

for


(2) $$\begin{array}{c} \text{muscle}\in \left\{\text{DIA},\text{EXT},\text{LEV},\text{SPL},\text{VL}\right\}\\ \text{group}\in \left\{\text{DM},\text{CO}\right\}\\ \text{i}\in \left\{1,2,\ldots 16\right\} \end{array}$$

The three spectral components MC1 , MC2 and  MC3 , represent vibrational properties of the skeletal muscle tissue and were used to identify its macromolecular constituents. The term  rmusclegroup,i  in Eq. (1) represents the remaining part that compensates for the difference from the normalised ATR-FTIR spectra as measured for the wet samples. The weights (concentrations) c1 , c2 , and c3 corresponding to the spectral components MC1 , MC2 and MC3 , respectively, were further elaborated statistically by reporting their mean values with the standard error of the mean (SEM). Two-way mixed ANOVA (with Greenhouse–Geisser correction) was performed for weights c1 , c2 , and c3 to analyse the statistical significance of the effects of skeletal muscle type and diabetes on macromolecular composition. We performed a *post-hoc* Tukey test to assess the significance of differences between muscles within the group and between DM and CO groups for a given muscle. The level of significance was 0.05.

### 2.4 Histochemical assays and data analysis

The histochemical analysis of skeletal muscle samples employed 10 μm thick serial transverse cryosections obtained using a Leica CM 1950 microtome (Leica Microsystems, Germany) thermostatically regulated at -25°C. Each section was mounted on a clean slide and examined prior to staining to verify the accuracy of the cross-sectional cuts. Haematoxylin- and eosin-stained slides were used to observe the general tissue morphology, following which semiquantitative methods were employed to assess the lipid, glycogen, and collagen content of the tissue based on previously published protocols (78–83). Sudan black B powder (Sigma-Aldrich Corp, St. Louis, MO, USA), which specifically stains neutral lipids black (78), was used for lipid staining. The Periodic Acid-Schiff (PAS) method described by McManus (81) was employed for staining polysaccharides, including glycogen. The staining of collagen bundles in tissue sections was performed using 0.1% Sirius red in saturated aqueous picric acid, as previously described by Junqueira et al. (83). The stained tissue sections were examined under a Nikon Eclipse 80i microscope (20x objective, numerical aperture: 0.50), which was equipped with an ODC-84 Kern-Sohndigital camera and VIS Pro microscope KERN OXM 902 software (Kern-Sohn, Germany) for image acquisition. A minimum of three randomly sampled fields of view free of artefacts were captured for each muscle section at a resolution of 5440 x 3648 pixels, using consistent settings for all similarly stained sections. Semiquantitative image analysis was performed with ImageJ (http://rsbweb.nih.gov/ij/) software, which can differentiate and precisely quantify areas stained with various colours by analysing and converting the pixels within the image into corresponding area values. The lipid content of the tissue was semiquantitatively assessed using ImageJ software, where the intensity of staining in greyscale mode was measured. The estimation of glycogen content was performed using ImageJ software, where the intensity of staining in the red channel obtained using the colour deconvolution plugin was measured. The mean grey value was calculated after converting the red channel to an 8-bit grayscale image (84). Completely white and completely black areas were assigned values of 0 and 255, respectively. The intensity of colour was expressed as relative intensity calculated as the ratio of measured mean grey value to 255. The collagen content index (defined as 100 times the ratio of the area of collagen-stained skeletal muscle tissue to the cross-sectional area of skeletal muscle tissue) was calculated using the same software by segmenting the red (collagen) and yellow (muscle fibres)-stained tissue with the thresholding and colour deconvolution plugin (82, 84). Two-way mixed ANOVA (with Greenhouse–Geisser correction) was performed for all three skeletal muscle indices (lipid, collagen and glycogen content) to analyse the statistical significance of the effects of skeletal muscle type and DM on macromolecular composition. The *post-hoc* Tukey test was performed to evaluate the significance of differences between muscles within the group and between DM and CO groups for a given muscle. The level of significance was 0.05. All investigative protocols, including muscle tissue sectioning, staining, and image acquisition and analysis, were performed in a blinded manner throughout the study.

## 3 Results

### 3.1 Skeletal muscle composition analysed by FTIR

**Figure 2:**
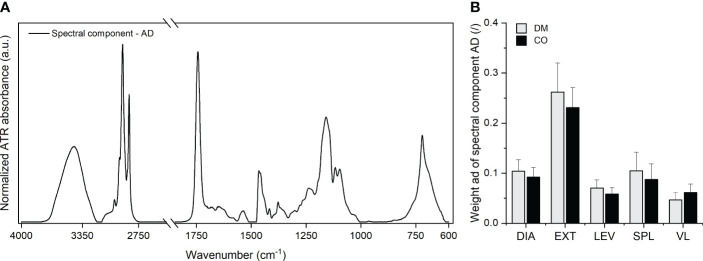
**(A)** Adipose tissue spectral component AD and **(B)** the corresponding weight ad obtained for five different muscles from 16 diabetic donors (DM group) and 16 non-diabetic donors (CO group) from the first MCR decomposition step. Skeletal muscles are abbreviated as follows: diaphragm (DIA), external intercostal muscles (EXT), levator scapulae muscle (LEV), splenius capitis muscle (SPL), vastus lateralis muscle (VL). Data in **(B)** are means with standard errors of the mean (SEM).

In the **Supplementary Figure 3** we show the spectra after removal of the body water spectral component BW in the form of an average and the dispersion of the spectra around the average. The images with the most dispersed data (the most pronounced grey shaded area in **Supplementary Figure 3**) in the regions 3500-2700 cm^-1^, 1800-1700 cm^-1^ and 1250-700 cm^-1^ indicate that this variability stems from variable amount of fats. Indeed, as given in subsections 2.2 and 2.3 of the Materials and methods section, in the first MCR decomposition step we obtained the spectral component AD , which is presumably attributed to the adipose tissue. This was a part of the samples that could not be mechanically removed from the skeletal muscle tissue during sample preparation. **Figure 2** shows this component and the corresponding weights, *ad*.

The absorption bands seen in **Figure 2A** correspond to the absorption bands observed in the spectra of various animal body fats, as shown in the work of Rohman and Che Man (85). In addition, the spectral component AD indicates the absorption peaks that are attributable to body water in adipose tissue (OH stretching band in the range between 3000-4000 cm^-1^), amide I and amide II bands of proteins, nucleic acids (low intensity bands in the range between 900-1000 cm^-1^). Here, the proportions between the intensities of the absorption bands reflect the proportions of these constituents characteristic of the composition of adipose tissue, as presented in the work of Stroh et al. (86). The weights $ad_{i}$ in **Figure 2B** determine the amount of adipose tissue in the analysed samples. The bar graphs indicate that the largest amount of adipose tissue was present in the EXT muscle samples. **Supplementary Figure 9** shows the corresponding scatterplots of *ad* and indicates that the EXT muscle samples had larger variance in the amount of adherent adipose tissue than other muscles. These trends are consistent with the challenges we faced in removing adipose tissue during sample preparation.

**Figure 3:**
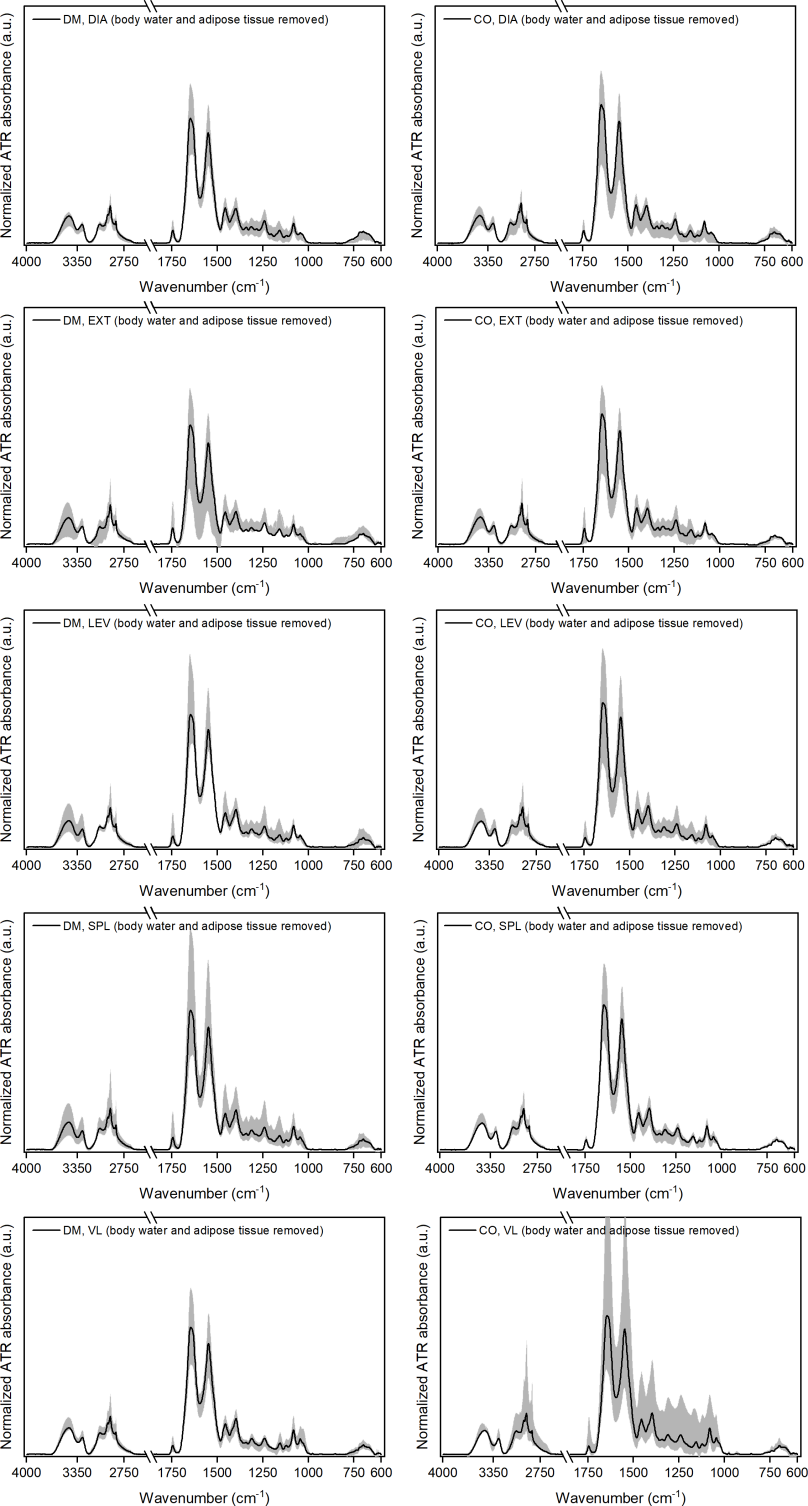
Normalised ATR-FTIR spectra after removing the weighted spectral components representing body water, *BW*, and adipose tissue, *AD* (i.e. MmuscleCgroup,i=muscleNSgroup,i−bwi·BW−adi·AD=musclec1group,i·MC1+musclec2group,i·MC2+musclec3group,i·MC3+musclergroup,i ) for five different muscles from 16 diabetic donors (DM group) and 16 non-diabetic donors (CO group). Diagrams in the left-hand column correspond to the DM group and in the right-hand column to the CO group. Skeletal muscles are abbreviated as follows: diaphragm (DIA), external intercostal muscles (EXT), levator scapulae muscle (LEV), splenius capitis muscle (SPL), vastus lateralis muscle (VL). The solid black line in each graph shows the average spectrum for each of the five skeletal muscles for both study groups. The grey shaded area represents the dispersion of the spectra, with the lower and upper contours of this area corresponding to the spectra that deviate the most from the average.

The MmuscleCgroup,i spectra obtained after removing the spectral components of body water and adipose tissue [see Eqs. (1) and (2)], are shown in **Figure 3**.

After removing the corresponding proportion of adipose tissue from the spectra, the dispersion of the spectra around the average becomes smaller (see grey shaded areas of individual diagrams in **Supplementary Figure 3**; **Figure 3** for comparison). The only exception here is the non-diabetic VL muscle, where the grey shaded area (dispersion of the data) remains wide, but we will see in continuation that this is due to the outlier related to the MC3 component.

To demonstrate the intermediate results after each decomposition step, **Supplementary Figure 4** shows the spectra after removing the weighted spectral components BW , AD and MC3 , and **Supplementary Figure 5** shows the spectra after removing the weighted spectral components BW , AD , MC3 and MC2 , i.e. the ${\;}_{\mathrm{muscle}}c1_{group,i}\cdot MC1+{\;}_{\mathrm{muscle}}r{group,i}_{\;}$ spectra. Comparing the corresponding individual plots in these two figures, it can be seen that the joint contribution of the MC1 and MC2 components has a relatively low dispersion (see relatively narrow grey shaded areas in **Supplementary Figure 4**), while the contributions of MC1 and MC2 separately appear to be widely dispersed (see wide grey shaded areas in **Supplementary Figure 5**).

**Figure 4:**
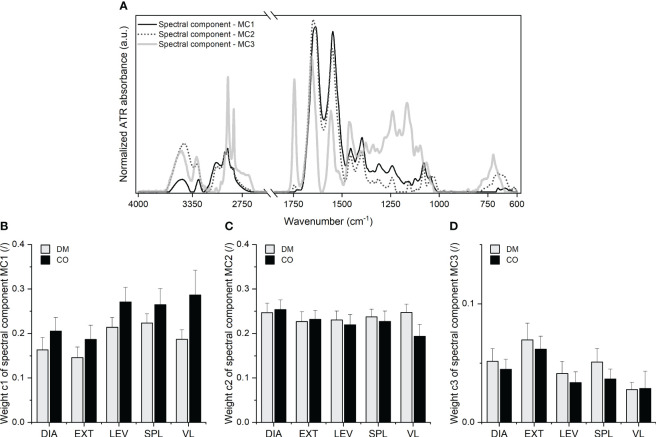
**(A)** Three spectral components, *MC*1, *MC*2 and *MC*3 with the corresponding **(B)** weight *c*1, **(C)** weight c2 and **(D)** weight c3 obtained for five different muscles from 16 diabetic donors (DM group) and 16 non-diabetic donors (CO group) from the second and third MCR decomposition steps. Skeletal muscles are abbreviated as follows: diaphragm (DIA), external intercostal muscles (EXT), levator scapulae muscle (LEV), splenius capitis muscle (SPL), vastus lateralis muscle (VL). Data in **(B–D)** are means with standard errors of the mean (SEM).

The final result of the decomposition, which reflects the biochemical profiles of skeletal muscle, is shown in **Figure 4** in the form of the spectral components MC1 , MC2 and MC3 with the corresponding weights c1 , c2 and c3 for further consideration.

The analysis of the spectral components shown in **Figure 4A** with their second derivatives enabled the identification of the absorption peak wavenumbers and the tentative assignment of vibrational bands. The second derivatives are shown in **Supplementary Figures 6**–**8**, with a list of wavenumbers in **Supplementary Tables 1**–**3** corresponding to the peaks of the second derivative. Tentative assignment of the peaks, i.e. the absorption bands, can be found below in **Table 1**.

**Table 1:** Tentative assignment of vibrational bands with the identification of the macromolecular constituents.

Spectral component	Macromolecules and their constituents represented by the spectral component	Tentative assignment of the most evident absorption bands characteristic of the macromolecular constituents represented by the spectral component
**MC1**	**Proteins**	• 1685 and 1630 cm^-1^: amide I (C=O stretching, C-N stretching, CNN deformation) of **β-sheet protein secondary structures** (49, 87–91) • 1651 cm^-1^: amide I (C=O stretching, C-N stretching, CNN deformation) of **α-helical protein secondary structures** (46, 49, 89, 92, 93) • 1547 cm^-1^: amide II (C-N stretching coupled with N-H bending) of **α-helical** and **β-sheet protein secondary** structures (46, 87, 93) • 1469, 1456, 1419, 1397 cm^-1^: CH_3_ and CH_2_ bending vibrations mostly of **protein side chains** (36, 87, 94–98) • 1342, 1310 cm^-1^: CH_2_ wagging modes and amide III (99) • 700-630 cm^-1^: amide IV O=C—N deformation and amide V N—H out-of-plane deformation (100) • 1516, 854 cm^-1^: **tyrosine ring vibrations** (87, 101–103) • 1172, 1157 cm^-1^: CO stretching mode of the C-OH groups of **serine, threonine, and tyrosine residues in proteins** (~1160 cm^-1^ - hydrogen-bonded CO group, and ~1170 cm^-1^ - non-hydrogen-bonded CO groups) (104–106) • 1242 cm^-1^: antisymmetric stretching of PO_2_^-^ group of **phosphorylated proteins** (107) • 1080 cm^-1^: symmetric stretching of PO_2_^-^ group of **phosphorylated proteins** (107) • 978 cm^-1^: symmetric stretching mode of dianionic phosphate monoesters of **phosphorylated proteins** (108)
	**Nucleic acids**	• 1367, 1046, 990 cm^-1^: **carbohydrates in nucleic acids** (109–112) • 1124 cm^-1^: stretching vibration of the skeletal structure of **ribose (RNA)** (112) • 1253, 1241, 1206 cm^-1^: antisymmetric stretching of PO_2_^-^ group of **nucleic acids** (104, 113) • 1080 cm^-1^: symmetric stretching of PO_2_^-^ group of **nucleic acids** (104, 113) • 978 cm^-1^: symmetric stretching mode of dianionic phosphate monoesters of **cellular nucleic acids** (104) • 936, 924 cm^-1^: **nucleic acids** (109)
**MC2**	**Proteins**	• 1688 and 1631 cm^-1^: amide I (C=O stretching, C-N stretching, CNN deformation) of **β-sheet protein secondary structures** (49, 87–91) • 1653 cm^-1^: amide I (C=O stretching, C-N stretching, CNN deformation) of **α-helical protein secondary structures** (46, 49, 89, 92, 93) • 1549, 1513 cm^-1^: amide II (C-N stretching coupled with N-H bending) of **α-helical** and **β-sheet protein secondary structures** (46, 87, 93) • 1467, 1456, 1420, 1397 cm^-1^: CH_3_ and CH_2_ bending vibrations mostly of **protein side chains** (36, 87, 94–98) • 1341, 1313, 1284 cm^-1^: CH_2_ wagging modes and amide III (99) • 720-630 cm^-1^: amide IV O=C—N deformation and amide V N—H out-of-plane deformation (100) •1155 cm^-1^: CO stretching mode of the C-OH groups of **serine, threonine, and tyrosine residues in proteins** (~1160 cm^-1^ - hydrogen-bonded CO group) (104–106) •1242 cm^-1^: antisymmetric stretching of PO_2_^-^ group of **phosphorylated proteins** (107) • 1081 cm^-1^: symmetric stretching of PO_2_^-^ group of **phosphorylated proteins** (107) • 978 cm^-1^: symmetric stretching mode of dianionic phosphate monoesters of **phosphorylated proteins** (108)
	**Glycogen**	• 1155, 1081, 1043, 1027, 938, 763 cm^-1^: vibrational bands characteristic of **glycogen** (87, 104, 114–117)
	**Nucleic acids**	• 1366, 1043, 995 cm^-1^: **carbohydrates in nucleic acids** (109–112) • 1122 cm^-1^: stretching vibration of the skeletal structure of **ribose (RNA)** (112) • 1242 cm^-1^: antisymmetric stretching of PO_2_^-^ group of **nucleic acids** (104, 113) • 1081 cm^-1^: symmetric stretching of PO_2_^-^ group of **nucleic acids** (104, 113) • 978 cm^-1^: symmetric stretching mode of dianionic phosphate monoesters of **cellular nucleic acids** (104) • 940 cm^-1^: **nucleic acids** (109)
	**Lipid intermediates**	• 1745 cm^-1^: C=O stretching of **lipid esters** (49, 71, 85) • 750-720 cm^-1^: CH_2_ rocking of **saturated fatty acids** (Coates, 2004) (118)
**MC3**	**Lipids**	• 3005 cm^-1^: olefinic -C=CH stretching vibration of **unsaturated fatty acids** (37, 85, 119) • 3000 - 2800 cm^-1^: CH_3_ and CH_2_ antisymmetric and symmetric stretching of **lipids** (37, 65, 85, 110, 119) • 1745 cm^-1^: C=O stretching of **lipid esters** (49, 71, 85) • 1465, 1456, 1438, 1417 cm^-1^: CH_3_ and CH_2_ bending vibrations mostly of **fatty acids** and **phospholipids** (36, 85, 87, 94, 120–122) and cis =C–H bending at 1417 cm^-1^ of **unsaturated fatty acids** (123) • 1404 cm^-1^: C=O symmetric stretching of COO^-^ groups of **fatty acids** (124–126) • 1378, 1340, 1319 cm^-1^: CH_3_ symmetric bending mostly of **fatty acids** and **phospholipids** (85, 114, 121, 122, 126) • 1167, 1143 cm^-1^: C-O-C bonds between the glycerol carbon and fatty acid ester carbon of **triglycerides** (85, 127) and at 1143 cm^-1^ C—OH bond of **membrane-bound oligosaccharide** (128) • 1118, 1098 cm^-1^: stretching vibration of the C–O ester groups (85, 123, 129) and at 1098 cm^-1^ PO_2_^-^ symmetric stretching of **phospholipids** (121, 122) • 1098, 1065 cm^-1^: small contribution of **phospholipids** vibrations (121, 122) • 973 cm^-1^: =C-H out-of-plane bending of **unsaturated fatty acids** (85, 124) and possibly with contribution of N^+^-(CH_3_)_3_ vibration of **phospholipids** (121, 122) • 940, 827, 816, 810, 772, 740 cm^-1^: vibrations of **fatty acids** and **phospholipids** (85, 121, 122, 130) • 721 cm^-1^: =C-H group bending of **unsaturated fatty acids** (131)
	**Collagen**	• 1658 cm^-1^: amide I in **collagen** (97, 132, 133) • 1557 cm^-1^: amide II in **collagen** (97, 132, 133) • 1455, 1340, 1280, 1240, 1205 cm^-1^: vibrational bands characteristic of **collagen** (97, 134–136); collagen amide III (1280, 1205 cm^-1^: CH_2_ wagging vibration, 1240 cm^-1^: C-N stretching, N-H bending vibrations and wagging vibrations of CH_2_ groups in the **glycine backbone** and **proline side chains**) (133) • 742, 701, 686 cm^-1^: amide IV O=C—N deformation and amide V N—H out-of-plane deformation (100) • 1065, 1035 cm^-1^: C-O stretching vibrations of the **carbohydrate residues** in **collagen** and **proteoglycans** (133) • 1516, 851 cm^-1^: **tyrosine ring** vibrations (87, 101–103) • 920, 875 cm^-1^: C–C stretching vibrations of **hydroxyproline** and **proline** characteristic of collagen (137)

As presented in **Table 1**, the multi-stage MCR decomposition into spectral components gives a relatively detailed insight into the macromolecular composition of skeletal muscle that can be obtained from the vibrational spectrum of the tissue sample measured in a single experiment. In general, components MC1 and MC2 are dominated by proteins (except for collagen), indicating variations in the ratios of the different secondary structures and the level of protein phosphorylation, as well as nucleic acids. In MC2 , a lipid ester band with lower intensity and an absorption range between 750-720 cm^-1^ also indicate the presence of lipid intermediates. The third spectral component MC3 is dominated by the vibrational absorption of lipid esters (mainly triglycerides, but also phospholipids) and collagen. Since in the spectral component MC3 certain absorption peaks are assigned exclusively to lipids and certain peaks exclusively to collagen, a higher/lower weight corresponding to MC3 simultaneously means a higher/lower proportion of lipids and a higher/lower proportion of collagen. On may note that all three spectral components contain information about proteins, but it is evident (especially from the amide I and II bands, their shape, frequencies and intensity ratios) that each spectral component reflects vibrational characteristics of proteins with specific structural properties. These hidden details, which can be revealed by spectral decomposition, provide a useful basis for the search for the possible structural changes in skeletal muscle proteins due to their impairment by diabetes. It is important to emphasise [as provided in Eq. (1)] that all analysed ATR-FTIR spectra have been described as a linear combination of the same spectral components obtained from the multi-stage decomposition, where the corresponding weights define the contribution of each spectral component to a given spectrum. In other words, the same spectral components for all analysed spectra reflect the same biomolecular profiles which have been contained in different proportions in the analysed samples. Therefore, the weights determine these proportions, i.e. the contribution of the macromolecular constituents represented by a given spectral component to the overall composition of the samples. In particular, the weights c1 , c2 and c3 are distinguished parameters that reflect the contribution of the macromolecular species represented by the spectral components MC1 , MC2 and MC3 in skeletal muscle tissue. Examination of the mean values of the weights c1 and c3 in **Figures 4B, D** indicates the differences in the macromolecular composition of the tissue between the diabetic group and the control group for a given muscle. Nevertheless, the two-way mixed ANOVA did not yield a statistically significant effect of the group, but did show statistical significance of the effect of muscle type on weights c1 (F_3.072, 92.15_ = 5.223; p = 0.0021) and c3 (F_3.320, 99.60_ = 6.847; p = 0.0002). **Supplementary Figures 10**–**12** additionally show the scatterplots for all three weights, where we can observe a lower variance of *c*1 for diabetic muscles compared to non-diabetic ones. The scatterplots in **Supplementary Figure 11** show a similar variance for c2 for diabetic and non-diabetic muscles. The variance of c3 for diabetic DIA, EXT and VL (when the outlier is excluded) muscles appears to be larger than for control muscles, while LEV and SPL muscles seem to have the same tendency, but it is less clear whether certain data should be considered as outliers. Further comments can be found in subsection 3.3 for comparison with the histochemical results.

### 3.2 Skeletal muscle composition analysed by histochemical assays

**Figure 5:**
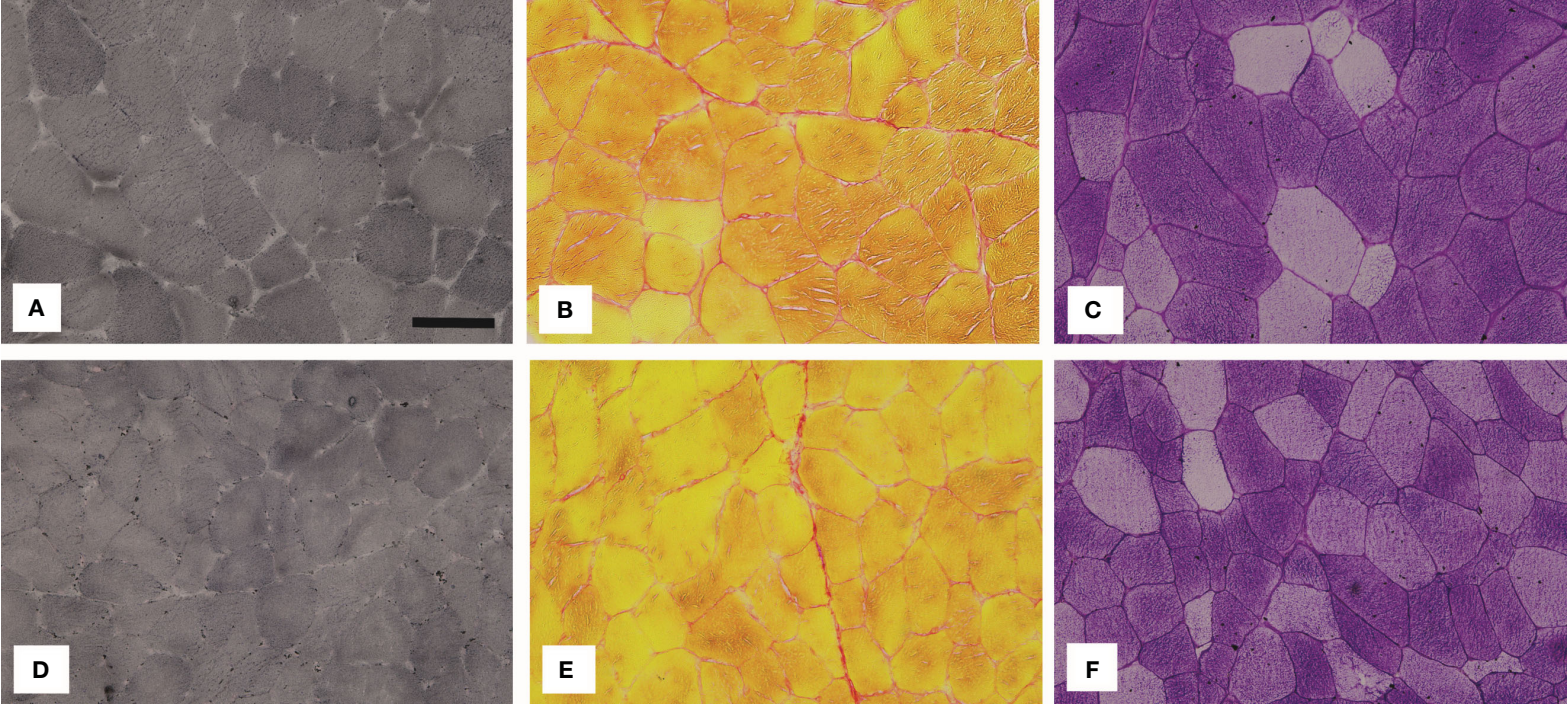
Representative images of the vastus lateralis muscle (VL) stained with **(A, D)** Sudan Black B (lipids), **(B, E)** Sirius red (collagen) and **(C, F)** PAS (glycogen). The images in the top row show the muscle VL of diabetic (DM) individuals and the images in the bottom row of non-diabetic (CO) individuals. The black bar in **(A)** represents 50 µm.

**Figure 6:**
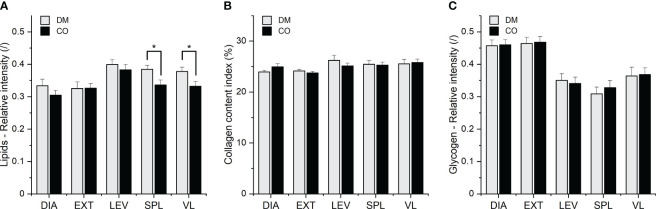
**(A)** Lipid, **(B)** collagen and **(C)** glycogen composition of five different muscles from 16 diabetic donors (DM group) and 16 non-diabetic donors (CO group) determined by histochemical analysis. Skeletal muscles are abbreviated as follows: diaphragm (DIA), external intercostal muscles (EXT), levator scapulae muscle (LEV), splenius capitis muscle (SPL), vastus lateralis muscle (VL). Data are means with standard errors of the mean (SEM); *p < 0.05, two-way mixed ANOVA.

**Figure 5** depicts representative images of histochemically stained sections, while **Figure 6** summarises the results of the histochemical analysis of the early postmortem macromolecular composition of the skeletal muscles in diabetic and non-diabetic individuals.

The two-way mixed ANOVA showed the statistical significance of the effect of the group (DM and CO) on lipid content (F_1, 30_ = 4.420; p = 0.0440), but not on collagen and glycogen content. There was also a statistically significant effect of muscle type on lipid content (F_2.092, 62.77_ = 8.396; p = 0.0005), collagen (F_2.781, 83.42_ = 3.523; p = 0.0211) and glycogen content (F_2.465, 73.95_ = 31.69; p< 0.0001) in different skeletal muscles. The *post-hoc* Tukey test revealed statistically significant differences between the diabetic (DM) group and non-diabetic (CO) group for muscles SPL and VL, as shown in **Figure 6A**. **Supplementary Figures 13**–**15** show the scatterplots for the three macromolecular species. When outliers are excluded, the variance in lipid amount appears to be greater in diabetic DIA and EXT muscles than in control muscles, whereas the variances are similar in diabetic and non-diabetic LEV, SPL and VL muscles. After excluding the outliers, one may also argue that the variances of collagen and glycogen amounts are similar for diabetic and control muscles, with the exception of LEV muscle, where the variance of collagen content is greater for diabetic than for non-diabetic muscle. Further comments can be found in the following subsection 3.3 for comparison with the spectroscopic results.

### 3.3 Comparison of the spectroscopic and histochemical results

In order to compare the two sets of results, we had to identify the outcomes of the spectroscopic analysis that matched the outcomes of the histochemical analysis, i.e. the lipid, collagen and glycogen content. Based on the assignments of the vibrational bands of the spectral components shown in **Table 1**, the lipid and collagen contents in the macromolecular composition of the muscles are controlled by the weight c3 and the glycogen content by the weight c2 . The qualitative comparison of the spectroscopic and histochemical results is given across the matched parameter pairs, i.e. histochemically determined lipid content compared to c3 , histochemically determined collagen content compared to c3 , histochemically determined glycogen content compared to c2 .

The scatterplots in **Supplementary Materials** point to the greater variance in the data obtained in spectral decomposition than in the data obtained in histochemical test. Nevertheless, the two approaches provide similar overall picture about the significance of disease status (diabetic or control), both revealing differences between the diabetic and non-diabetic groups, mainly in the form of increased intramuscular lipid content. Although the spectroscopic approach showed no statistically significant differences between the groups in terms of lipid content, there is a general trend towards an increased amount of lipids in diabetic muscles compared to control muscles (see weight *c*3 in **Figure 4D**; **Supplementary Figure 12**). This is mainly consistent with the corresponding trends in histochemical analysis (see **Figure 6A**; **Supplementary Figure 13**). As far as collagen and glycogen content are concerned, both methods agree that up to this point there are no significant differences between the groups with regard to these two macromolecular components.

Regarding the influence of muscle type on tissue composition in general, both methods agree that muscle type has an influence on lipid content (histochemistry: p = 0.0005; ATR-FTIR via *c*3: p = 0.0002) and collagen content (histochemistry: p = 0.0211; ATR-FTIR via *c*3: p = 0.0002), but differ with respect to glycogen content (histochemistry: p< 0.0001; ATR-FTIR via *c*2: no statistical significance of muscle type effect on glycogen content). However, when comparing the muscles with each other, the spectroscopic analysis seems to show a somewhat different picture than the histochemistry. For example, the mean value of *c*3 (which determines the amount of lipids) and its variance seem to be higher for the EXT muscle (see **Figure 4D**; **Supplementary Figure 12**) than for other muscles. On the other hand, the bar graphs and scatterplots representing the lipids analysed in the histochemical test do not show the same (see **Figure 6A**; **Supplementary Figure 13**).

The observed discrepancies between the outcomes of the spectroscopic approach and the histochemical analysis could primarily be due to differences in sample preparation, the form and amount of the sample analysed and the sample treatment during the histochemical analysis.

In addition, we analysed relatively small sample sets and are aware of the natural variability of the metabolic phenotype of human skeletal muscle. Accordingly, we remain cautious about further statistical details and definitive conclusions about DM-induced changes in tissue composition. Nevertheless, we have developed the key methodological steps to obtain numerous compositional details from the vibrational spectra that can be transferred to large sample sets relevant for commenting on DM-induced alteration in skeletal muscle biomolecular phenotype.

## 4 Discussion

The information in a single ATR-FTIR spectrum, derived from the spectral components and their corresponding weights, can provide a very valuable insight into the macromolecular composition of the tissues. Spectral decomposition yields a set of spectral components, which reflect a set of distinct chemical profiles, i.e. particular macromolecular species, which are common to all analysed spectra. As shown in **Table 1**, the amide I and amide II bands of the α-helical and β-sheet protein secondary structures present in the spectral components MC1 and MC2, and the structures belonging to collagen represented by MC3, suggest the changes in the overall protein structural pattern. The changing proportions of particular protein structures, due either to different muscle types or to the effects of DM, can be tracked by the weights c1, c2 and c3 of the spectral components. Similarly, the degree of phosphorylation of proteins, elevated or decreased levels of certain amino acids, nucleic acids, glycogen, lipid intermediates can be assessed from the weights c1 and c2 and, using the weight c3, the proportions of lipids, phospholipids and collagen. These spectroscopic analyses reflect detailed insights into the potential diabetic alterations in skeletal muscle metabolic phenotype, such as changes in protein expression and post-translational modifications, intramyocellular lipid deposition, impaired glycogen synthesis and impaired mitochondrial function. Therefore, the weights obtained from the spectral decomposition can serve as the main parameters for comparing diabetic and non-diabetic muscles and for analysing the correlations between the altered proportions of particular macromolecular species.

To comment on the discrepancies between the spectroscopic and histochemical results, we would like to argue that the former reflect a more realistic picture of composition. Our arguments are based on the fact that in the spectroscopic measurements we used a larger amount of the tissue sample, which was kneaded well to distribute the constituents as evenly as possible, in contrast to the histochemical tests where individual sections were used for analysis. Additionally, the possible effects of the staining procedure and the biases in the assessment of the stained localised areas belonging to a specific molecular species under investigation should be considered. On the other hand, the use of wet samples for FTIR spectroscopy could be a potential weakness. The large amount of body water in the tissue absorbs a large amount of the energy emitted by the infrared source in the FTIR experiment. Therefore, the absorption intensities of the solid part of the tissue are relatively low compared to the absorption intensities of the water, which may affect the dispersion of the spectra of the solid part of the tissue to a certain extent. Nevertheless, it is important to work with wet samples in order to exclude possible effects of drying the samples, which can strongly alter the structural properties of the proteins.

Despite numerous studies addressing skeletal muscle myopathy as a possible cause and/or consequence of DM, the understanding of the interactions remains incomplete. While most research have focused on the specific molecular mechanism, we believe that methodological approaches that provide insight into more macromolecular indicators simultaneously are the most efficient way to obtain a comprehensive picture and a deeper understanding of the molecular mechanisms of disease and their interactions. The methodology we have proposed in this article, using FTIR spectroscopy and the resourceful spectral decomposition approach, directly follows and supports this aspect.

We are aware that our pilot study uses relatively small sample sets and it is therefore too early to draw definitive conclusions about DM-induced changes in the composition of human skeletal muscle, although the results already suggest certain trends. However, the most important contribution expected from this preliminary study has been the development of an alternative, simple and efficient methodology to study the macromolecular composition of skeletal muscle tissue. The established methodological framework will ensure the feasibility of a large-scale study with statistically representative sample sizes, which are essential for obtaining robust data on the DM-related macromolecular changes in skeletal muscle. We believe that our spectroscopic approach can make an important contribution to DM research by providing a tool for monitoring disease progression in terms of its effects on skeletal muscle composition and the interrelationships between macromolecular alterations in tissue.

## 5 Conclusion

In summary, this paper proposes an alternative methodology for examining macromolecular constituents of biological tissue based on information in the vibrational spectrum. The approach was developed on a pilot sample set of five different skeletal muscles from 16 diabetic and 16 non-diabetic human donors. We have shown that with appropriate spectral decomposition steps we can obtain much more information about the macromolecular composition of skeletal muscle than with several different histochemical tests. Within the proposed methodological framework we have:

- established detailed protocols for skeletal muscle sample preparation and the implementation of the spectroscopic experiments to minimise the impact of these procedures on the measured FTIR spectra;
- laid the foundations for the multi-stage spectral decomposition of FTIR spectra into the spectral components for the simultaneous identification of various macromolecular species and their contributions to the overall composition of skeletal muscle;
- identified the characteristic spectral absorption bands in order to identify the individual macromolecular species represented by a particular spectral component;
- identified the parameters for comparing the contribution of macromolecular species to the overall composition of different skeletal muscles in diabetic and non-diabetic subjects and for analysing their correlations;
- demonstrated the versatility and efficiency of the proposed spectroscopic approach in comprehensively revealing subtle changes in the composition of skeletal muscle.

On this basis, we are confident that we have brought our methodology to a level from which it can be successfully transferred to a large-scale study that allows for statistically representative analyses and suitable conditions to search for statistically significant changes in skeletal muscle composition due to DM.

## Data availability statement

The raw data supporting the conclusions of this article will be made available by the authors, without undue reservation.

## Ethics statement

The studies involving humans were approved by the Republic of Slovenia National Medical Ethic Committee (Permit No.: 0120-536/2019/4). The human samples used in this study were acquired from skeletal muscle tissue of deceased adult (≥ 18 years) male bodies within 24 hours postmortem. The skeletal muscle tissue was harvested during standard autopsy procedures at the Institute of Forensic Medicine, University of Ljubljana, Slovenia. The tissue samples and the data obtained were pseudonymized, and only the authorized forensic medicine specialist had access to the data of the deceased included in the study. Written informed consent for participation was not required from the participants or the participants' legal guardians/next of kin in accordance with the national legislation and institutional requirements. The ethical policies are regulated by the national directive »PRAVILNIK o pogojih in načinu opravljanja mrliško pregledne službe«; Uradni list RS, pp. 7430 / No. 99 / 22. 7. 2022; https://www.uradni-list.si/_pdf/2022/Ur/u2022099.pdf, according to which the standard autopsy procedures are carried out on the basis of the coroner's decision (Article 10 of the directive) and not on the basis of the informed consent of the participants or the participants' legal guardians/next of kin. For standard autopsies, the recommendations of the Council of Europe are followed, i.e., »Recommendation No. R (99) 3 of the Committee of Ministers to Member States on harmonization of medico-legal autopsy rules« and the »Appendix to Recommendations No. R (99) 3«; https://www.coe.int/t/dg3/healthbioethic/texts_and_documents/RecR(99)3.pdf.

## Author contributions

BZ: Conceptualization, Data curation, Formal analysis, Investigation, Methodology, Project administration, Software, Supervision, Validation, Visualization, Writing – original draft. CKU: Data curation, Investigation, Methodology, Writing – review & editing. MEAA: Data curation, Formal analysis, Investigation, Methodology, Writing – original draft. AA: Investigation, Methodology, Writing – review & editing. EC: Conceptualization, Funding acquisition, Resources, Supervision, Writing – review & editing. JG: Conceptualization, Funding acquisition, Methodology, Resources, Supervision, Writing – review & editing. AŠ: Data curation, Investigation, Methodology, Writing – review & editing. NU: Conceptualization, Data curation, Formal analysis, Investigation, Methodology, Supervision, Writing – review & editing.
