# Supplementary material for: Exploration of macromolecular phenotype of human skeletal muscle in diabetes using infrared spectroscopy
Source: Front Endocrinol (Lausanne). 2023 Dec 21;14:1308373. doi: 10.3389/fendo.2023.1308373 (PMC10769457; doi:10.3389/fendo.2023.1308373)
Supplement: Supplementary file 1 [file DataSheet_1.docx]

Supplementary Material

# Supplementary Figures and Tables

**

**

**Supplementary Figure 1.** Bar graphs with scatterplots showing (**A**) age and (**B**) body mass index (BMI) of the DM and CO donors. Bar graph data show means with standard errors of the mean (SEM).

|  | **Supplementary Figure 2.** Normalised ATR-FTIR spectra ${}_{muscle}{{NS}_{group,i}}$ for five different muscles from 16 diabetic donors (DM group) and 16 non-diabetic donors (CO group). Diagrams in the left-hand column correspond to the DM group and in the right-hand column to the CO group. Skeletal muscles are abbreviated as follows: diaphragm (DIA), external intercostal muscles (EXT), levator scapulae muscle (LEV), splenius capitis muscle (SPL), vastus lateralis muscle (VL). The solid black line in each graph shows the average spectrum for each of the five skeletal muscles for both study groups. The grey shaded area represents the dispersion of the spectra, with the lower and upper contours of this area corresponding to the spectra that deviate the most from the average. |
| --- | --- |

|  | **Supplementary Figure 3.** Normalised ATR-FTIR spectra after removing the weighted spectral component representing body water, $BW$ (i.e. ${}_{muscle}{{NS}_{group,i}}-{bw}_{i}\cdot BW$) for five different muscles from 16 diabetic donors (DM group) and 16 non-diabetic donors (CO group). Diagrams in the left-hand column correspond to the DM group and in the right-hand column to the CO group. Skeletal muscles are abbreviated as follows: diaphragm (DIA), external intercostal muscles (EXT), levator scapulae muscle (LEV), splenius capitis muscle (SPL), vastus lateralis muscle (VL). The solid black line in each graph shows the average spectrum for each of the five skeletal muscles for both study groups. The grey shaded area represents the dispersion of the spectra, with the lower and upper contours of this area corresponding to the spectra that deviate the most from the average. |
| --- | --- |

|  | **Supplementary Figure 4.** Normalised ATR-FTIR spectra after removing the weighted spectral components representing body water, $BW$, the adipose tissue component, $AD$, and the $MC3$ spectral component (i.e. ${}_{muscle}{{NS}_{group,i}}-{bw}_{i}\cdot BW-{ad}_{i}\cdot AD-{}_{muscle}{{c3}_{group,i}}\cdot MC3={}_{muscle}{{c2}_{group,i}}\cdot MC2+ {}_{muscle}{{c1}_{group,i}}\cdot MC1+{}_{muscle}{r_{group,i}}$) for five different muscles from 16 diabetic donors (DM group) and 16 non-diabetic donors (CO group). Diagrams in the left-hand column correspond to the DM group and in the right-hand column to the CO group. Skeletal muscles are abbreviated as follows: diaphragm (DIA), external intercostal muscles (EXT), levator scapulae muscle (LEV), splenius capitis muscle (SPL), vastus lateralis muscle (VL). The solid black line in each graph shows the average spectrum for each of the five skeletal muscles for both study groups. The grey shaded area represents the dispersion of the spectra, with the lower and upper contours of this area corresponding to the spectra that deviate the most from the average. |
| --- | --- |

|  | **Supplementary Figure 5.** Normalised ATR-FTIR spectra after removing the weighted spectral components representing body water, $BW$, the adipose tissue component, $AD$, the $MC3$ spectral component, and the $MC2$ spectral component (i.e. ${}_{muscle}{{NS}_{group,i}}-{bw}_{i}\cdot BW-{ad}_{i}\cdot AD-{}_{muscle}{{c3}_{group,i}}\cdot MC3-{}_{muscle}{{c2}_{group,i}}\cdot MC2= {}_{muscle}{{c1}_{group,i}}\cdot MC1+{}_{muscle}{r_{group,i}}$) for five different muscles from 16 diabetic donors (DM group) and 16 non-diabetic donors (CO group). Diagrams in the left-hand column correspond to the DM group and in the right-hand column to the CO group. Skeletal muscles are abbreviated as follows: diaphragm (DIA), external intercostal muscles (EXT), levator scapulae muscle (LEV), splenius capitis muscle (SPL), vastus lateralis muscle (VL). The solid black line in each graph shows the average spectrum for each of the five skeletal muscles for both study groups. The grey shaded area represents the dispersion of the spectra, with the lower and upper contours of this area corresponding to the spectra that deviate the most from the average. |
| --- | --- |



 Supplementary Figure 6. Second derivative of the spectral component $MC1$ with numbered peaks (listed below in Supplementary Table 1 with the corresponding wavenumbers).

Supplementary Table 1. Wavenumbers corresponding to the numbered peaks in the second derivative of the spectral component $MC1$ (see Supplementary Figure 6 above).

| **Peak number**  **(see Supplementary Figure 6)** | **Corresponding**  **wavenumber**  **(cm^-1^)** |
| --- | --- |
| **1** | 1685 |
| **2** | 1651 |
| **3** | 1630 |
| **4** | 1547 |
| **5** | 1516 |
| **6** | 1469, 1456, 1419, 1397 |
| **7** | 1342, 1310 |
| **8** | 1241, 1206 |
| **9** | 1172, 1157 |
| **10** | 1124 |
| **11** | 1080 |
| **12** | 1046, 990 |
| **13** | 978 |
| **14** | 936 |
| **15** | 854 |
| **16** | 700-630 |



 Supplementary Figure 7. Second derivative of the spectral component $MC2$ with numbered peaks (listed below in Supplementary Table 2 with the corresponding wavenumbers).

Supplementary Table 2. Wavenumbers corresponding to the numbered peaks in the second derivative of the spectral component $MC2$ (see Supplementary Figure 7 above).

| **Peak number**  **(see Supplementary Figure 7)** | **Corresponding**  **wavenumber**  **(cm^-1^)** |
| --- | --- |
| **1** | 1745 |
| **2** | 1688 |
| **3** | 1653 |
| **4** | 1631 |
| **5** | 1549 |
| **6** | 1513 |
| **7** | 1467, 1456, 1420, 1397 |
| **8** | 1341, 1313, 1284 |
| **9** | 1242 |
| **10** | 1155 |
| **11** | 1122 |
| **12** | 1081 |
| **13** | 1043 |
| **14** | 1027 |
| **15** | 978 |
| **16** | 763 |
| **17** | 750-720 |



 Supplementary Figure 8. Second derivative of the spectral component $MC3$ with numbered peaks (listed below in Supplementary Table 3 with the corresponding wavenumbers).

Supplementary Table 3. Wavenumbers corresponding to the numbered peaks in the second derivative of the spectral component $MC3$ (see Supplementary Figure 8 above).

| **Peak number**  **(see Supplementary Figure 8)** | **Corresponding**  **wavenumber**  **(cm^-1^)** |
| --- | --- |
| **1** | 3005 |
| **2** | 3000-2800 |
| **3** | 1745 |
| **4** | 1658 |
| **5** | 1557 |
| **6** | 1516 |
| **7** | 1465 |
| **8** | 1404 |
| **9** | 1378, 1340, 1319 |
| **10** | 1280, 1240, 1205 |
| **11** | 1167, 1143 |
| **12** | 1118, 1098 |
| **13** | 1065, 1035 |
| **14** | 973 |
| **15** | 920, 875 |
| **16** | 851 |
| **17** | 830740 |
| **18** | 721 |





**Supplementary Figure 9.** Bar graph with scatterplots showing weight $ad$ obtained for five different muscles from 16 diabetic donors (DM group) and 16 non-diabetic donors (CO group), corresponding to the spectral component $AD$ from the first MCR decomposition step. Skeletal muscles are abbreviated as follows: diaphragm (DIA), external intercostal muscles (EXT), levator scapulae muscle (LEV), splenius capitis muscle (SPL), vastus lateralis muscle (VL). Bar graph data show means with standard errors of the mean (SEM).





Supplementary Figure 10. Bar graph with scatterplots showing weight $\boldsymbol{c1}$ obtained for five different muscles from 16 diabetic donors (DM group) and 16 non-diabetic donors (CO group), corresponding to the spectral component $\boldsymbol{MC1}$ from the third MCR decomposition step. Skeletal muscles are abbreviated as follows: diaphragm (DIA), external intercostal muscles (EXT), levator scapulae muscle (LEV), splenius capitis muscle (SPL), vastus lateralis muscle (VL). Bar graph data show means with standard errors of the mean (SEM).





Supplementary Figure 11. Bar graph with scatterplots showing weight $\boldsymbol{c2}$ obtained for five different muscles from 16 diabetic donors (DM group) and 16 non-diabetic donors (CO group), corresponding to the spectral component $\boldsymbol{MC2}$ from the third MCR decomposition step. Skeletal muscles are abbreviated as follows: diaphragm (DIA), external intercostal muscles (EXT), levator scapulae muscle (LEV), splenius capitis muscle (SPL), vastus lateralis muscle (VL). Bar graph data show means with standard errors of the mean (SEM).





Supplementary Figure 12. Bar graph with scatterplots showing weight $\boldsymbol{c3}$ obtained for five different muscles from 16 diabetic donors (DM group) and 16 non-diabetic donors (CO group), corresponding to the spectral component $\boldsymbol{MC3}$ from the second MCR decomposition step. Skeletal muscles are abbreviated as follows: diaphragm (DIA), external intercostal muscles (EXT), levator scapulae muscle (LEV), splenius capitis muscle (SPL), vastus lateralis muscle (VL). Bar graph data show means with standard errors of the mean (SEM).





Supplementary Figure 13. Bar graph with scatterplots showing lipid composition of five different muscles from 16 diabetic donors (DM group) and 16 non-diabetic donors (CO group) determined by histochemical analysis. Skeletal muscles are abbreviated as follows: diaphragm (DIA), external intercostal muscles (EXT), levator scapulae muscle (LEV), splenius capitis muscle (SPL), vastus lateralis muscle (VL). Bar graph data show means with standard errors of the mean (SEM).





**Supplementary Figure 14.** Bar graph with scatterplots showing collagen composition of five different muscles from 16 diabetic donors (DM group) and 16 non-diabetic donors (CO group) determined by histochemical analysis. Skeletal muscles are abbreviated as follows: diaphragm (DIA), external intercostal muscles (EXT), levator scapulae muscle (LEV), splenius capitis muscle (SPL), vastus lateralis muscle (VL). Bar graph data show means with standard errors of the mean (SEM).





Supplementary Figure 15. Bar graph with scatterplots showing glycogen composition of five different muscles from 16 diabetic donors (DM group) and 16 non-diabetic donors (CO group) determined by histochemical analysis. Skeletal muscles are abbreviated as follows: diaphragm (DIA), external intercostal muscles (EXT), levator scapulae muscle (LEV), splenius capitis muscle (SPL), vastus lateralis muscle (VL). Bar graph data show means with standard errors of the mean (SEM).
